# Laryngopharyngeal reflux disease in the Greek general population, prevalence and risk factors

**DOI:** 10.1186/s12901-015-0020-2

**Published:** 2015-12-21

**Authors:** Nikolaos Spantideas, Eirini Drosou, Anastasia Bougea, Dimitrios Assimakopoulos

**Affiliations:** Athens Speech Language and Swallowing Institute, 10 Lontou Street, Glyfada, Athens, 16675 Greece; Athens Speech Language and Swallowing Institute, 37 Oinois Street, Glyfada, Athens, 16674 Greece; Athens Speech and Language Institute, 1 Griva Digeni Street, Agios Dimitrios, Athens, 17342 Greece; Department of Otorhinolaryngology, University Hospital of Ioannina, Medical School of Ioannina University, 51 Napoleontos Zerva Street, Ioannina, 45332 Greece

**Keywords:** Reflux, Gastroesophageal reflux, Laryngopharyngeal reflux, Epidemiologic study, Risk factors

## Abstract

**Background:**

To assess the prevalence of laryngopharyngeal reflux (LPR) in the Greek general population and its risk factors.

**Methods:**

Questionnaire based epidemiological, adult participants’ survey. The Reflux Symptom Index (RSI) was used for the assessment of LPR prevalence. The RSI questionnaire was completed by 340 (183 male and 157 female) randomly selected subjects. Subjects with RSI score ≥13 were considered as LPR patients and those with RSI score <13 were considered as non LPR subjects.

**Results:**

The prevalence of LPR in the general Greek population was found to be 18.8 % with no statistically significant difference between the two genders (p > 0.05). The age group of 50–64 years showed the higher prevalence rate. Tobacco smoking and alcohol consumption were found to be related with LPR. No reported concomitant disease or medication was found to be related with LPR.

**Conclusions:**

LPR prevalence in the Greek general population was found to be 18.8 %. Tobacco smoking and alcohol consumption were found to be related with LPR.

## Background

LPR is defined as the retrograde movement of gastric contents into the larynx and pharynx leading to a diversity of upper aero digestive tract symptoms [1].

LPR has a significant negative impact on patient’s quality of life [2]. LPR is an underdiagnosed entity in otolaryngology and its actual prevalence and predisposing factors in the community have not been established. Potential risk factors and co-morbidities for LPR remain unknown. Data concerning LPR prevalence in Greece are lacking.

In 2002, Belafsky et al developed the Reflux Symptom Index (RSI), a self-administered nine-item questionnaire, designed to assess various symptoms related to LPR. Each item is scaled from 0 (no problem) to 5 (severe problem), with a maximum score of 45 indicating the most severe symptoms. An RSI ≥ 13 is considered abnormal and strongly indicative of LPR [3].

Since the introduction of RSI, many studies have shown the reliability and consistency of the method in various populations throughout the globe, establishing the method as a very useful diagnostic tool in every day practice [4–6]. Feng GJ et al have found that laryngopharyngeal pH monitoring and RSI scoring have the same value in diagnosing laryngopharyngeal reflux disease (LPRD) [7].

The primary aim of this study was to assess the prevalence of LPR in the general adult Greek population using RSI as the diagnostic screening tool. Secondary aims of the study were to identify any predisposing or associated factors for developing LPR.

## Methods

The study was carried out in the general Greek population during the period from September to November 2013.

A random sample (n = 1.000) of adults living in Athens (500 people) and in rural Greek areas (500 people) was initially approached through an “alert” telephone. During the communication, the scope of the study was explained and permission to send the questionnaire to the subjects’ address was obtained. The participants were randomly selected through the telephone catalogue of Athens City and telephone catalogues of randomly selected rural areas using a Table of random numbers generated for the study. Five different investigators performed the calls ten days before sending the questionnaire. Of the 1000 subjects who were approached, 450 accepted to participate in the study and provided their personal details (name - address). Only one person per family has to fill the sent questionnaire. In the envelope that was sent to the participants a more detailed explanation for the scope of the study, detailed instructions for filling out the questionnaire, an informed consent and a prepaid envelope were included, so that subjects could easily send back the filled-in questionnaire as well as the signed informed consent at no cost for them. Three hundred fifty individuals returned the questionnaires (189 or 54 % from Athens and 161 or 46 % from rural areas).

Data related to LPR symptoms were gathered through a questionnaire containing the validated Greek version of RSI. Additional questions concerning demographic data (age and gender), behavioral characteristics (smoking status and alcohol consumption) concomitant diseases and concurrent medication were also included in the posted questionnaire.

Inclusion criterion for our study was age since the main scope of our study was to asses LPR prevalence in the general adult population. In this regard, only subjects >18 year old were included in the study. Subjects with pre-existing gastro-esophageal reflux and those taking anti-reflux medications were also included in the study. Exclusion criteria for participation in the study were current upper respiratory tract infections and known laryngopharyngeal malignancies.

For the purpose of this study LPR diagnosis was based on RSI score ≥ 13 as proposed by Belafsky et al [3].

The study protocol was approved by the Scientific Committee and Review Board of Athens Speech, Language and Swallowing Institute. Informed consent was obtained from all participants prior to inclusion in the study.

Statistical tests were performed using the IBM SPSS Statistics 20 software.

Variables of the analysis were: Demographic parameters, smoking and alcohol habits, health background (concomitant diseases), concomitant medication and the reflux symptom index.

The independent samples of Student’s test were used to compare the RSI values in the patients with LPR and in subjects without LPR which were used as control group.

The study sample size was calculated based on the assumption that LPR prevalence is higher than 15 %

Statistical tests used for the statistical analysis were:

- Significance level for all hypothesis testing (*p*-value) was 0.05
- Correlations between the levels of the RSI were performed using the Spearman’s Rho correlation coefficient test.
- The prevalence of LPR was estimated using a minimum cut-off score of ≥ 13 on the RSI as proposed by Belafski et al. [3]

We assessed the prevalence of LPR in the general Greek population using the RSI. In this regard, the Greek version of the validated RSI questionnaire was given to 450 subjects from different parts of Greece (50 % from urban areas and 50 % from rural areas) to be completed. Eventually 350 completed questionnaires were collected.

## Results

The questionnaire was given to 450 subjects. Three hundred and fifty subjects (response rate 77.8 %) returned completed questionnaires. In 10 out of the 350 returned questionnaires, critical information like gender and age were lacking. Thus 340 (183 male and 157 female) duly completed questionnaires were appropriate for statistical analysis. Demographic data and patients’ behavioral characteristics are shown in Table 1. The mean age of the participants was 46.86 ± 14.54 years. Most participants belonged to the age group of 35–49 (131 subjects) while the age groups > 80 and < 20 were poorly represented (3 subjects > 80 year and 2 subjects < 20 year).

**Table 1:** Demographic data and behavioral characteristics of study participants

*Variable*	*Group*	*Number*	*Percent*
Gender	Female	157	46.2
	Male	183	53.8
Total		340	100.0
Age (in years) Mean (±SD): 46.86 (±14.54)	<20	2	0.6
	20–34	71	20.9
	35–49	131	38.5
	50–64	88	25.9
	65–79	45	13.2
	> = 80	3	0.9
Total		340	100.0
Smoking	Yes	174	51.2
	No	166	48.8
Total		340	100
Duration of smoking (years) Mean (±SD): 18.23 (±8.6)	1–5	7	4.1
	5–10	29	17.2
	10–15	39	23.1
	15–20	34	20.1
	20–25	30	17.8
	25–30	9	5.3
	>30	21	12.4
Total		169	100
Number of cigarettes per day Mean (±SD): 20.51 (±11.83)	1–9	18	13.7
	10–19	38	29.0
	20–29	42	32.1
	30–39	17	13.0
	40–49	13	9.9
	50–59	1	0.8
	60+	2	1.5
Total		131	100
Drinker	No	239	70.3
	Yes	101	29.7
Total		340	100
Number of drinks per day	1–2	72	71.3
	3–4	17	16.8
	5–6	11	10.9
	9–10	1	1.0
Total		101	100

One hundred seventy four (51.2 %) participants were smokers with mean number of cigarettes per day 20.5 and mean duration of smoking 18.23 ± 8.6 years for male and 17.02 ± 8.0 years for female, with no statistically significant difference between the two genders (*t*-test >0,05).

One hundred and one subjects (29.7 %) drank alcohol regularly (68.3 % male and 31.7 % female). Mean alcohol consumption per day was 2.43 ± 1.62 units for males and 2.17 ± 1.32 units for females with no statistical significance between the two genders (*t*-test > 0.05). The most commonly reported alcoholic drinks were wine (30) followed by beer (21) and whisky (18).

One hundred forty two (41.8 %) participants reported one or more diseases. The reported diseases were: cardiovascular 50 (35.2 %), gastrointestinal 25 (17.6 %), musculoskeletal 15 (10.6 %), respiratory 10 (7.0 %), thyroidopathy 9 (6.3 %), anemia 3 (2.1 %) and other diseases 30 (21.1 %). Among the reported gastrointestinal diseases, 5 cases were gastroesophageal reflux disease (GERD), 13 dyspepsia, 5 gastritis and 2 duodenal ulcers.

One hundred and thirty seven (40.3 %) participants reported use of one or more medication for the concomitant diseases. The reported medications were: antihypertensive 43 (31.4 %), anticholesterol 19 (13.9 %), antiulcerants 19 (13.9 %), [15 PPIs (proton pump inhibitors) and 4 H_2_ antagonists], antidiabetics 11 (8.0 %), anti-asthmatics/COPD (chronic obstructive pulmonary disease) 11 (8.0 %), antihypothyroidism 9 (6.6 %), antiosteoporotics 7 (5.1 %) [5 calcium carbonate and 2 bisphosphonates], non steroidal anti-inflammatory drugs 4 (1,2 %) and other medications 18 (13.1 %).

Two hundred sixty six subjects (78.2 %) reported one or more symptoms included in the RSI. The most commonly reported symptoms were No 9 “Heartburn, chest pain, indigestion, or stomach acid coming up” (52 %) and No 2 “Clearing your throat” (48.2 %) (Table 2).

**Table 2:** Frequency of reported symptoms included in reflux symptom index by the participants of the study

Symptom	Number	Percent
1. Hoarseness or a problem with your voice	131	38,5
2. Clearing your throat	164	48,2
3. Excess throat mucus or postnasal drip	130	38,2
4. Difficulty swallowing food, liquids, or pills	99	29,1
5. Coughing after you ate or after lying down	110	32,3
6. Breathing difficulties or choking episodes	108	31,8
7. Troublesome or annoying cough	105	30,9
8. Sensation of something sticking in your throat or a lump in your throat	138	40,6
9. Heartburn, chest pain, indigestion, or stomach acid coming up	177	52

Sixty four subjects (18.8 %) out of the 340 participants of our study presented an RSI ≥13 and were considered as patients with LPR, compared to 276 subjects with an RSI < 13 who were considered to be subjects without LPR. The mean RSI score in patients with LPR was 24.8 ± 8.0 compared to the subjects without LPR the mean RSI of which was 2.3 ± 3.2 (p < 0.001) (Table 3).

**Table 3:** Mean score and standard deviation of the reflux symptom index items in patients with LPR and in non LPR subjects

Group	RSI1	RSI2	RSI3	RSI4	RSI5	RSI6	RSI7	RSI8	RSI9	Total RSI
LPR										
Mean	3,1598	3,6201	2,8217	1,8081	2,1246	3,1041	1,9204	2,8788	3,3224	24,76
SD	1,2624	1,5578	1,1601	1,3196	1,4513	1,3143	1,4723	1,1786	1,5923	8,0032
Non LPR										
Mean	0,2218	0,3832	0,4689	0,2125	0,1987	0,0989	0,1375	0,3137	0,2718	2,3207
SD	0,3726	0,4576	0,7238	0,3806	0,9086	0,8076	0,7178	0,4463	0,6534	3,2261
*P* value LPR vs Non LPR	<0,001	< 0,001	< 0,001	< 0,001	< 0,001	< 0,001	< 0,001	< 0,001	< 0,001	< 0,001

RSI1: Hoarseness or a problem with your voice, RSI2: Clearing your throat, RSI3: Excess throat mucus or postnasal drip, RSI4: Difficulty swallowing food, liquids, or pills, RSI5: Coughing after eating or after lying down, RSI6: Breathing difficulties or choking episodes, RSI7: Troublesome or annoying cough, RSI8: Sensations of something sticking in your throat or a lump in your throat, RSI9: Heartburn, chest pain, indigestion, or stomach acid coming up

Spearman’s Rho correlations analysis showed that all the pairs between the 9 items of the RSI were correlated, meaning that if a subject responded positively to one item there was a high probability to respond positively to the other item.

Based on the findings of our study the prevalence of LPR in the Greek general population was found to be 18.8 %. The LPR prevalence for males was 19.7 % and for females 17.8 % with no statistically significant difference between the two genders (*t*-test, p > 0.05).

Most subjects with LPR (RSI ≥13) belonged to the age groups of 50–64 year (40.6 %) and 35–49 (34.4 %). These two age groups represented 75 % of the LPR cases encountered in the general Greek population. No LPR cases reported in ages >80 and <20 but this may be due to the very small sample size of these two particular age groups.

Statistical analysis did not show any relation between LPR and any of the reported diseases nor LPR and reported medications (Chi-square test > 0.05 for both cases). The lack of such findings has to be accepted with reservations and not as conclusive due to the limited number of reported diseases and medications, and since the primary aim of this study was not to assess these two parameters.

**Fig. 1:**
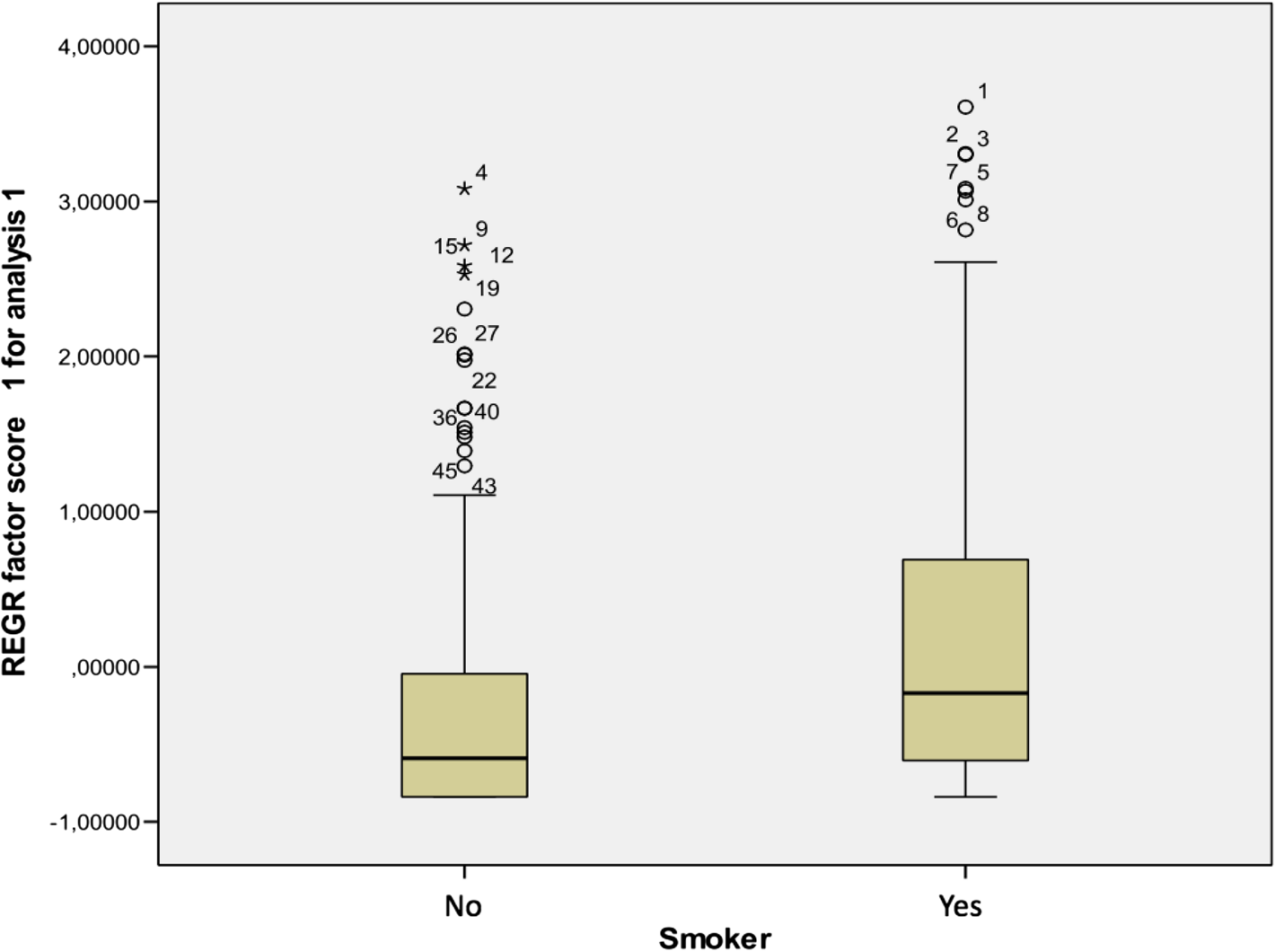
Box-plot of RSI factor score between smokers and non smokers

**Fig. 2:**
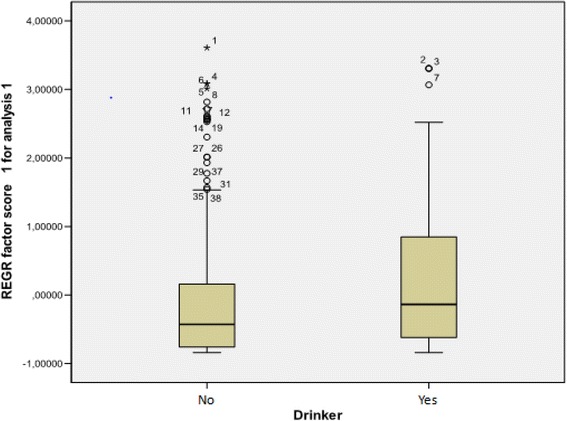
Box-plot of RSI factor score between drinkers and non drinkers

A correlation was found between LPR and smoking and alcohol consumption. Factor analysis was used to assess a potential association between the Factor’s Score and the information available for every person. It was concluded that alcohol drinkers and nondrinkers have a statistically significant difference in their mean factor score, as well as smokers compared to nonsmokers (*t*-test, p-value < 0.001 and p-value = 0.006 respectively. The direction of this association is shown in the box plots (Figs. 1 and 2).

We should be aware that the smokers of this study tend to consume alcohol more often than non-smokers. For that reason we cannot be sure which of the two, tobacco or alcohol consumption has an effect on increasing the average score of the RSI.

## Discussion

LPR remains a controversial topic with inconsistent data concerning its epidemiology, etiology, diagnosis and management [8].

It is difficult to estimate the prevalence of LPR in the general population since there is not an easy and generally accepted diagnostic method available for large scale epidemiological studies [9]. It has been reported that up to 10 % of patients presenting to an otolaryngologist’s office and more than 50 % of patients with hoarseness are patients with reflux related disease [10, 11]. LPR episodes have been reported by 30–50 % of the normal control [12, 13] and the prevalence of LPR in the general population has been reported to vary between 7.1 % [14] to 64 % [9]. The big difference in the reported LPR prevalence is mainly attributed to the differences in the methods used by each investigator as well as to the absence of a generally adopted definition of LPR.

The need for an easily administered and generally accepted diagnostic method for early detection of LPR patients is crucial, considering that LPR is better at predicting the presence of esophageal adenocarcinoma than typical gastroesophageal reflux symptoms [15], and that LPR is related to laryngopharyngeal carcinoma [16].

There is not a reliable known prevalence of LPR symptoms in the Greek population and thus the objectives of this study were to use a validated tool, the RSI in Greek, to identify LPR symptoms.

In this study subjects scoring RSI ≥ 13 are presumed to be LPR patients and those with RSI < 13 were presumed to be LPR free subjects.

According to the findings of this study, the prevalence of LPR in the general Greek population was found to be 18.8 %. No significant difference was observed between males and females in the prevalence of LPR. The age group where LPR prevalence was reported more frequently was 50–64 (40.2 %). Lowden et al demonstrated that 26.5 % of patients attending a general practice in UK had an RSI >10 [17]. Kamani T et al. have shown that 30 % of the UK general population have an RSI > 10 [18]. A study conducted in Greece using the RSI as a diagnostic tool for LPR has found the prevalence of LPR to be 8.5 % in the Greek population. However, that study did not refer to the general population since the participants were mainly ambulatory patients who were visiting primary care centers for various chronic diseases or patients’ escorts [19]. Another drawback of the previously mentioned study was the exclusion of subjects with certain diseases like irritable bowel syndrome (IBS), peptic ulcer disease, major psychiatric illnesses and those using non steroidal anti-inflammatory drugs (NSAIDs), conditions that are all well known to have a higher LPR prevalence [18, 20]. In addition, the gender make-up was not well balanced and male participants represented only 36.3 % of the study population vs 63.7 % female. Although this study did not find a significant difference in LPR prevalence between men and women in agreement with finding from other studies [18, 21], some other investigators have found LPR prevalence to be much higher in males [22, 23]. The different LPR prevalence rate obtained by the above mentioned studies, which used RSI as diagnostic tool for LPR diagnosis, reflects the different methodology each investigator used regarding LPR definition and population selection.

It is important to mention the high frequency of LPR related symptoms reported by the participants of our study. Two hundred sixty out of the total 340 (78.2 %) reported one or more symptom included in the RSI. Most common reported symptoms were RSI9 “Heartburn, chest pain, indigestion, or stomach acid coming up” (52 %) and RSI2 “Clearing your throat” (48.2 %). The high frequency of RSI9, especially in the middle aged people of the general population, is also in line with the findings of other investigators [18, 24].

No relation between LPR and reported diseases nor LPR and medication was found. However these findings could not be considered as conclusive due to the small number of reported concomitant diseases and to the small number of medications.

A correlation was found between LPR and smoking and alcohol consumption. Lin CC et al reported a correlation between total RSI and smoking as well as alcohol drinking with certain RSI items [4]. Kamani T et al did not find any association between LPR related symptoms and smoking or alcohol consumption [18]. It should be noted that in our study the smokers tend to consume alcohol more often than non-smokers. That’s why we cannot be sure which of the two, smoking or alcohol habits, has an effect on increasing the average score of the RSI nor which causes the other. Kamani T et al have found alcohol not to be a risk factor for LPR-related symptoms [18]. Controversy regarding the effect of alcohol exists not only for LPR, but also for GERD, as the results of different studies are diverse and contradictory. Despite the controversies regarding the effect of smoking and drinking on LPR, the recommendation of lifestyle modifications for the treatment of LPR include smoking cessation and limiting alcohol intake [25].

To the best of our knowledge, our study is the first that has been designed to assess the prevalence of LPR in the general population using the RSI score of ≥13 as the criterion for LPR diagnosis. Similar studies coming from different countries and populations can give us a more clear view on LPR prevalence. So that the findings of future studies are comparable we propose the cut-off point of RSI ≥13 to be the base for LPR diagnosis and the sample to refer to pure general population.

A limitation of this study could be the lack of comparison between the applied method and a method with higher specificity (flexible endoscopy or ambulatory 24-h double-probe pH monitoring), but on one hand these methods are invasive and costly and are not suitable for large scale epidemiological studies and on the other hand this comparison has already been done in other studies and has proved similar validity of the two methods [7]. Another limitation of the study could be the small sample size of concomitant diseases and medications that did not permit us to reach a confident conclusion regarding the relationship between LPR and the above mentioned factors.

## Conclusions

LPR prevalence in the general Greek population assessed by RSI was found to be 18.8 %. Tobacco smoking and alcohol consumption were found to be related with LPR. RSI is an easy and useful tool in daily clinical practice not only for the diagnosis and management of LPR but also for epidemiological studies.

## Availability of data and materials

All data and materials related to this study are kept in corresponding author’s office and are available on request.

## Abbreviations

LPR: Laryngopharyngeal reflux
LPRD: Laryngopharyngeal reflux disease
GERD: Gastroesophageal reflux disease
RSI: Reflux symptom index
PPIs: Proton pump inhibitors
COPD: Chronic obstructive pulmonary disease
IBS: Irritable bowel syndrome
NSAIDs: Non steroidal anti-inflammatory drugs
